# Alveolar macrophages support interferon gamma-mediated viral clearance in RSV-infected neonatal mice

**DOI:** 10.1186/s12931-015-0282-7

**Published:** 2015-10-05

**Authors:** Katherine M. Eichinger, Loreto Egaña, Jacob G. Orend, Erin Resetar, Kacey B. Anderson, Ravi Patel, Kerry M. Empey

**Affiliations:** Department of Pharmacy and Therapeutics, Center for Clinical Pharmaceutical Sciences, University of Pittsburgh School of Pharmacy, Pittsburgh, PA USA

**Keywords:** Neonate, RSV, Lung, IFNγ, Alveolar macrophage

## Abstract

**Background:**

Poor interferon gamma (IFNγ) production during respiratory syncytial virus (RSV) is associated with prolonged viral clearance and increased disease severity in neonatal mice and humans. We previously showed that intra-nasal delivery of IFNγ significantly enhances RSV clearance from neonatal lungs prior to observed T-lymphocyte recruitment or activation, suggesting an innate immune mechanism of viral clearance. We further showed that alveolar macrophages dominate the RSV-infected neonatal airways relative to adults, consistent with human neonatal autopsy data. Therefore, the goal of this work was to determine the role of neonatal alveolar macrophages in IFNγ-mediated RSV clearance.

**Methods:**

Clodronate liposomes, flow cytometry, viral plaque assays, and histology were used to examine the role of alveolar macrophages (AMs) and the effects of intra-nasal IFNγ in RSV infected neonatal Balb/c mice. The functional outcomes of AM depletion were determined quantitatively by viral titers using plaque assay. Illness was assessed by measuring reduced weight gain.

**Results:**

AM activation during RSV infection was age-dependent and correlated tightly with IFNγ exposure. Higher doses of IFNγ more efficiently stimulated AM activation and expedited RSV clearance without significantly affecting weight gain. The presence of AMs were independently associated with improved RSV clearance, whereas AM depletion but not IFNγ exposure, significantly impaired weight gain in RSV-infected neonates.

**Conclusion:**

We show here for the first time, that IFNγ is critical for neonatal RSV clearance and that it depends, in part, on alveolar macrophages (AMs) for efficient viral clearing effects. Early reductions in viral burden are likely to have profound short- and long-term immune effects in the vulnerable post-natally developing lung environment. Studies are ongoing to elucidate the pathologic effects associated with early *versus* delayed RSV clearance in developing neonatal airways.

## Background

Neonates, more than older children and adults have an increased risk for bronchiolitis and pneumonia, due in part, to the anti-inflammatory environment that supports post-natal lung development. Respiratory syncytial virus (RSV) is the most common respiratory virus in neonates and young children worldwide causing an estimated 3.4 million lower respiratory tract infections and approximately 200,000 deaths annually [1]. Nearly all children are infected with RSV by three years of age, but not all children develop severe disease [2, 3]. In addition to known risk factors associated with disease progression, including low birth weight, premature birth, and congenital heart disease, [4] recent clinical studies by Devincenzo’s group and others have tied high RSV titers to increased severity of disease and/or length of hospital stay [5–8].

The conditions present when a T cell encounters antigen for the first time are essential to subsequent immunity, often determining the nature of the response to infection. Alveolar macrophages (AMs) play an important role in establishing these initial conditions through cytokine production, recruitment of lymphocytes, and antimicrobial activity. However, these functions vary significantly in neonates compared to older children and adults and are often further influenced by invading pathogens. Though infants are prone to Th2-type cytokine responses left over from fetal life, they are capable of Th1-type immunity [9]. Other factors known to contribute to neonatal Th2 immunity are low levels of mature T cells and IFNγ, which when exposed to high burdens of viral antigen lead to Th2 immunity, producing such cytokines as IL-4, IL-5, and IL-13 [10, 11]. By regulating the cytokine environment through early, in vivo delivery of IFNγ, studies in neonatal models of murine leukemia virus have shown that immune responses can be shifted to a Type I pathway with enhanced viral clearance [12].

Interferon gamma (IFNγ) is a potent type II interferon known to stimulate direct antimicrobial effects among macrophages including the up-regulation of antigen processing and presentation [13]. IFNγ-stimulated macrophages secrete TNFα, IL-12 and other inflammatory cytokines, which facilitate the trafficking of T cells and NK cells to the site of inflammation [13]. Though its limited production in the early months after birth is thought to protect post-natal lung development, [14] elevated levels of IFNγ in the serum are associated with milder RSV disease, whereas detection of Th2-type cytokines, IL-4 and IL-5, are typically associated with greater disease severity [15–17]. Polymorphisms in IFNγ gene expression during neonatal RSV infection are linked to increased severity of illness, duration of stay in the intensive care unit (ICU), and frequency of otitis media [18]. Moreover, influenza- *versus* RSV-infected neonates produce markedly more IFNγ, suggesting that reduced production is likely linked, at least in part, to RSV infection [19]. Autopsy data of lung sections taken from human neonates who died with severe RSV infection show insufficient recruitment of Th1 and Th2 cytokine-producing lymphocytes, including CD4 and CD8 T cells as well as natural killer (NK) cells [19, 20], suggesting that impaired recruitment of IFNγ-producing cells are associated with increased disease severity.

In mouse models of RSV infection, local delivery of IFNγ has demonstrated both acute and long-term benefits in neonatal RSV infection. These include faster viral clearance as well as protection against RSV-mediated airway hyper-responsiveness (AHR) [21, 22]. This study aimed to determine if AMs can be therapeutically stimulated in a neonatal mouse model of RSV infection using inhaled IFNγ to enhance initial viral clearance, promote T cell immunity, and improve functional outcomes. Our lab has published data showing that neonatal mice produce negligible IFNγ in response to RSV with a corresponding delay in viral clearance compared to IFNγ-producing adults [23]. We further show that activation of AMs and pulmonary DCs are significantly enhanced upon delivery of intranasal IFNγ with expedited RSV clearance early in infection without eliciting weight loss commonly associated with the systemic delivery of IFNγ [23, 24]. Despite enhanced activation of innate immunity following i.n. IFNγ, the recruitment of CD4 and CD8 T cells was unexpectedly reduced in RSV-infected neonatal mice compared to age-matched controls [23].

## Materials and methods

### Ethics

This study was carried out in strict accordance with the recommendations in the Guide for the Care and Use of Laboratory Animals of the National Institutes of Health. Mice were housed at The University of Pittsburgh Division of Laboratory Animal Resources. These animal experiments were approved by The University of Pittsburgh Institutional Animal Care and Use Committee (IACUC), approved protocol number 14023340 and mice were handled according to IACUC guidelines. All efforts were made to minimize animal suffering.

### Mice and viral preparation

Pathogen-free breeder BALB/cJ breeder mice and IFNγ receptor knock out (IFNγR KO) mice were purchased from The Jackson Laboratory (Bar Harbor, ME) at 5–7 weeks of age and maintained in pathogen-free facilities. Females were bred as previously described, [23] and pups from the resultant pregnancies were treated experimentally at 2–7 days of age, as mice less than 7 days of age are considered neonates. Additional pathogen-free BALB/cJ female and male mice were purchased from The Jackson Laboratory at 8 weeks of age for experimental and control purposes as described below. Line 19 RSV was provided by Dr. Martin Moore, Emory University, Atlanta, GA. RSV line 19 and viral lung titers were prepared as previously described [23]. Briefly, RSV was passed through 4 rounds of plaque purification; after a single plaque was isolated, it was propagated in HEp-2 cells (American Type Culture Collection), then titered by standard hematoxylin-eosin (H&E) plaque assay, as previously described [25]. Viral stocks were snap-frozen and stored at −80 °C. Viral stocks and HEp-2 cell lines were routinely monitored for mycoplasma and other contaminants using the Plasmo Test Mycoplasma Detection Kit (InvivoGen) and the LookOut® Mycoplasma PCR Detection Kit according to manufacturer’s instructions. Lung titers were determined, as previously described, within one month of sterile removal and storage of infected lungs at −80 °C [25].

### Clodronate liposome delivery

To deplete alveolar macrophages, clodronate liposomes (Clip) (5 μl/g of 5 mg/ml suspension) were administered intranasally (i.n.) to 2-day-old mice under 2 % isoflurane anesthesia beginning day −1 post-infection, then daily until the mice were euthanized for sample collection. On days 1, 3, and 5 when IFNγ was co-administered, the doses of CLip and rIFNγ were separated by ≥ 6 h.

### Interferon gamma treatment

Recombinant-murine interferon gamma (IFNγ) (16 ng/g or 60 ng/g, Peprotech, NJ) was administered i.n. to 2–7 day old mice under 2 % isoflurane anesthesia on 1, 3, and 5 days post-infection. The total volume administered to adults was 50 μl and 10–20 μl to neonates. For the pharmacokinetic experiment, a single dose of 16 ng/g was administered i.n. to RSV-naïve mice at time zero and they were subsequently euthanized using 100 % isoflurane at 0, 0.5, 1, 2, 4, 6, 8, 12, 18, 24 and 48 h post-dose.

### RSV infection

Neonatal mice were infected with 5 × 10^5^ pfu/g to 3 × 10^6^ pfu/g of RSV L19 as previously described; [26] mock infected controls received identical volumes of cell lysate (supernatant from lysed cells that remained uninfected) or PBS. Animals were anesthetized prior to infection using 2 % isoflurane. Left lung was collected and snap frozen in alcohol/dry ice for subsequent quantification of lung titers, as previously described [25]. Bronchoalveolar lavage (BALF) and first wash were harvested and stored separately for flow cytometry or cytokine analysis, respectively. Right lungs were collected and immediately processed for analysis using flow cytometry or cytokine analysis as described below.

### Real-time polymerase chain reaction

Left lungs were snap frozen in liquid nitrogen for qRT-PCR as previously described [23]. Briefly, mRNA was harvested using Mini Qiagen Kit (Life Technologies, NY) and quantified using a NanoDrop® spectrophotometer (Invitrogen, NY). The mRNA was reverse transcribed to cDNA using a Superscript III First-strand synthesis Supermix for qRT-PCR kit (Life Technologies, NY) and quantified on a 7500 ABI Fast RTPCR system (Life Technologies). Pre-mixed Taqman primer and probes using a Fam/Tamara reporter/quencher combination were purchased from ABI specific for Gob5A. Results are represented as a relative increase from media only or from media + IFNγ, using the delta, delta ct method indicating fold change over the house keeping gene (GAPDH). Data are compared to control wells treated with media only using a paired *T*-test for 3–4 wells per group per mouse.

### Flow cytometry

Flow cytometry was used to evaluate surface protein expression in BALF and lung digest (LD) as previously described [23]. Right lungs were enzyme digested as previously described [27]. Briefly, lung tissue was minced, incubated with DNase and collagenase for 1 h at 37 °C, then pushed through a 70 micron mesh screen. RBCs were then removed from both BALF and LD with a hypertonic lysis buffer, cells were counted, and non-specific binding was blocked with anti-CD16/32 (BD Biosciences). Staining was performed with murine-specific fluorochrome-conjugated antibodies and fixed in 0.5–1 % paraformaldehyde prior to analysis with an LSRII or LSR Fortessa flow cytometer (BD Biosciences) within 12 h. Within the population of large granular cells, CD11b-PerCpCy™5.5 and CD11c-APC (BD Biosciences, San Jose, CA) or CD11c-PECy7 (Biolegend, San Diego, CA) were compared in dot plot quadrants, AMs were defined as CD11c + CD11b-, and activation of AMs was determined by the expression of major histocompatibility complex (MHC) class II (MHC II; I-a^d^)-FITC.. Data was analyzed using FlowJo software (Tree Star Inc. Ashland, OR).

### Histopathology

At 8 days post-infection (dpi), right lungs were gravity filled (25 cm from meniscus to catheter) with 10 % formalin after flushing the respiratory system with phosphate buffered saline (PBS). Lungs were preserved for at least 48 h in 10 % formalin at 4 °C. The lungs were paraffin-embedded and stained processed at the Transplant Pathology Research Laboratory of University of Pittsburgh (Pittsburgh, PA). Terminal deoxynucleotidyl transferase dUTP nick end labeling (TUNEL) and periodic acid-Schiff (PAS) stains were used to identify cells undergoing apoptosis and mucus accumulation, respectively. Slides were examined and quantified by two individuals blinded to treatment group. PAS-staining was scored according to previously published methods [23].

### Collection and processing of biological samples

Right lungs were collected, weighed and immediately snap-frozen in liquid nitrogen. Samples were stored at -80^0^ C until they were processed for protein quantification and cytokine analysis as previously described [28]. Briefly, frozen lungs were homogenized in cold Tissue Protein Extraction Reagent (T-PER, Thermo Scientific) and protease inhibitor (HALT Protease Inhibitor Cocktail, Thermo Scientific) (1 mL T-PER + 10 μl HALT protease inhibitor cocktail used for every 100 μg of frozen lung tissue), then centrifuged at 9000 g for 10 min at 4^0^ C. The supernatant was collected and total protein was quantified by bicinchoninic acid assay (BCA assay, Thermo Scientific Pierce) per manufacturer’s instructions. The remaining supernatant was stored at −80 °C for cytokine analysis. IFNγ concentrations in lung homogenate and first wash samples were analyzed by a murine multiplex cytokine kit (Bio-Rad, Hercules, CA). Homogenized lung samples were diluted to a total protein concentration of 500 μg/ml using a 1:1 mixture of T-PER and sample diluent (provided by manufacturer). Samples of first wash were added directly to the plate without dilution. The plates were read using a Luminex® 200™ Total System machine (Luminex Corp, Austin, Tx); data was analyzed using the LDS1.7 Software.

### Pharmacokinetic analysis

Lung IFNγ concentration-time data after a single i.n. IFNγ dose was analyzed by non-compartmental analysis (NCA). The terminal elimination rate constant, k_el_, was estimated by log-linear regression of at least three time points visually assessed to be in the terminal phase of each lung concentration-time plot. The terminal phase (elimination) half-life, t_1/2_, was calculated as ln2/k_el_. The area under the lung concentration *versus* time curve, AUC_0→∞,_ was also calculated. Using the linear trapezoidal rule, [29] AUC_last→∞_ was extrapolated to infinity by calculating C_last_/k_el_. The AUC from time zero to infinity was calculated as the sum of AUC_last_ and C_last_/k_el_. Other pharmacokinetic parameters calculated include IFNγ total body clearance, estimated as Dose/AUC. The estimated neonatal dose capable of achieving adult AUC values was calculated by dividing the adult AUC_0→∞_ by neonatal total body clearance. The new neonatal dose was then calculated on a per gram basis by dividing dose by the average weight of a 3–5 day-old neonate.

### Statistical analysis

Data are expressed as the mean ± SEM of at least three mice per group. Statistical analysis was performed using GraphPad Prism 5 software (La Jolla, CA). A two-way ANOVA was used to compare differences among data collected at multiple time points between ≥ 2 neonatal groups or between neonatal groups receiving multiple treatments followed by a Bonferroni post-test. For analysis of multiple groups of neonates at a single time point a 1-way ANOVA was used with a Tukey-Kramer post-test or a Kruskal Wallis test for nonparametric data. A two-way repeated measures ANOVA was used to compare differences in weight gain for neonates in the AM depletion study.

## Results

### Signaling through the IFNγR is required for efficient viral clearance

**Fig. 1:**
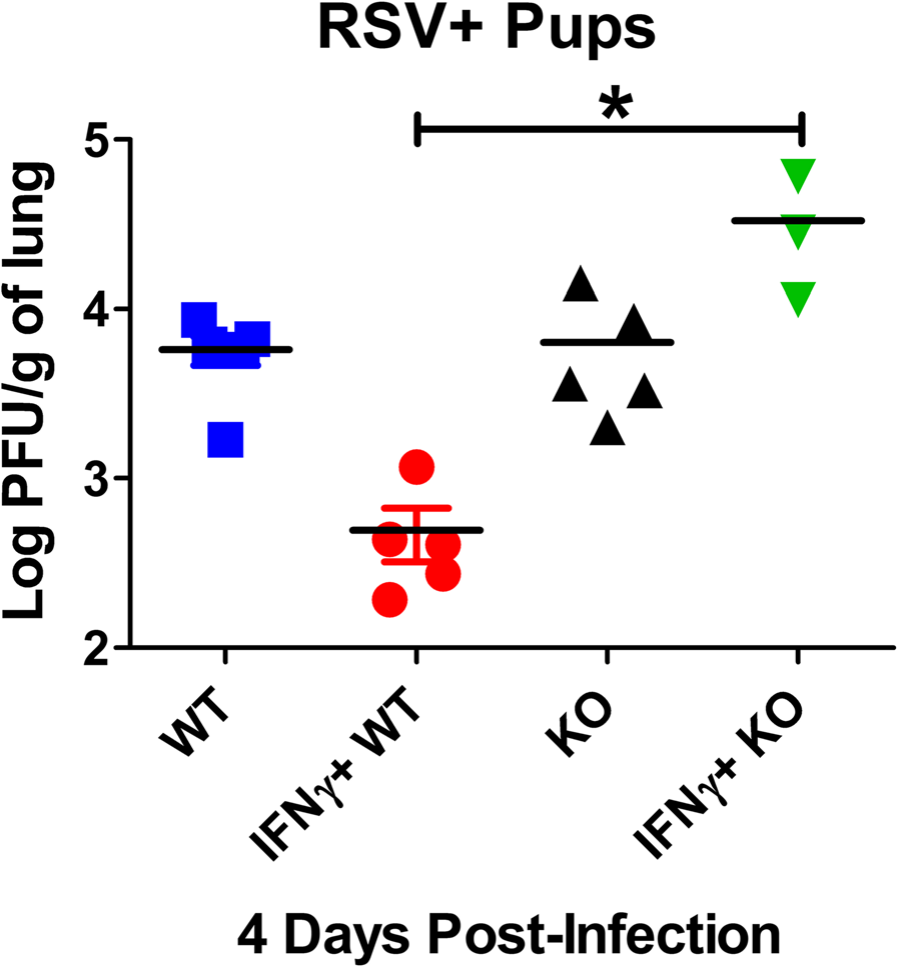
Inhaled IFNγ contributes to RSV clearance. Pup BALB/c or IFNγRKO mice were infected with RSV L19. Pups were treated with 16 ng/g of IFNγ or equal volumes of PBS on 1 and 3 dpi. Left lungs were harvested to quantify RSV using H&E plaque assays. * indicates *p* < 0.05 using a Kruskal-Wallis non-parametric analysis with Dunn’s multiple comparison test. Data represent the means and individual replicates for ≥ 3 mice per group and 2 separate experiments

We first sought to determine whether an absence of IFNγ signaling would alter viral clearance. To test this, viral clearance capacity was determined in IFNγR knock-out (IFNγRKO) mice compared to wild-type (WT) pups, both on a BALB/c background, with or without intranasal (i.n.) delivery of IFNγ as described in the methods (Fig. 1). Viral quantification by viral plaque assay at 4 dpi demonstrates that IFNγ does not kill RSV directly, but requires interaction with the IFNγR to elicit its anti-viral effects. No difference in viral titers between KO and WT pups confirms negligible production of endogenous IFNγ in early RSV infection, while reduced RSV titers in IFNγ-treated, WT controls suggests that intact IFNγ receptors are required for efficient RSV clearance.

### Age-dependent activation of AMs can be enhanced by i.n. IFNγ

**Fig. 2:**
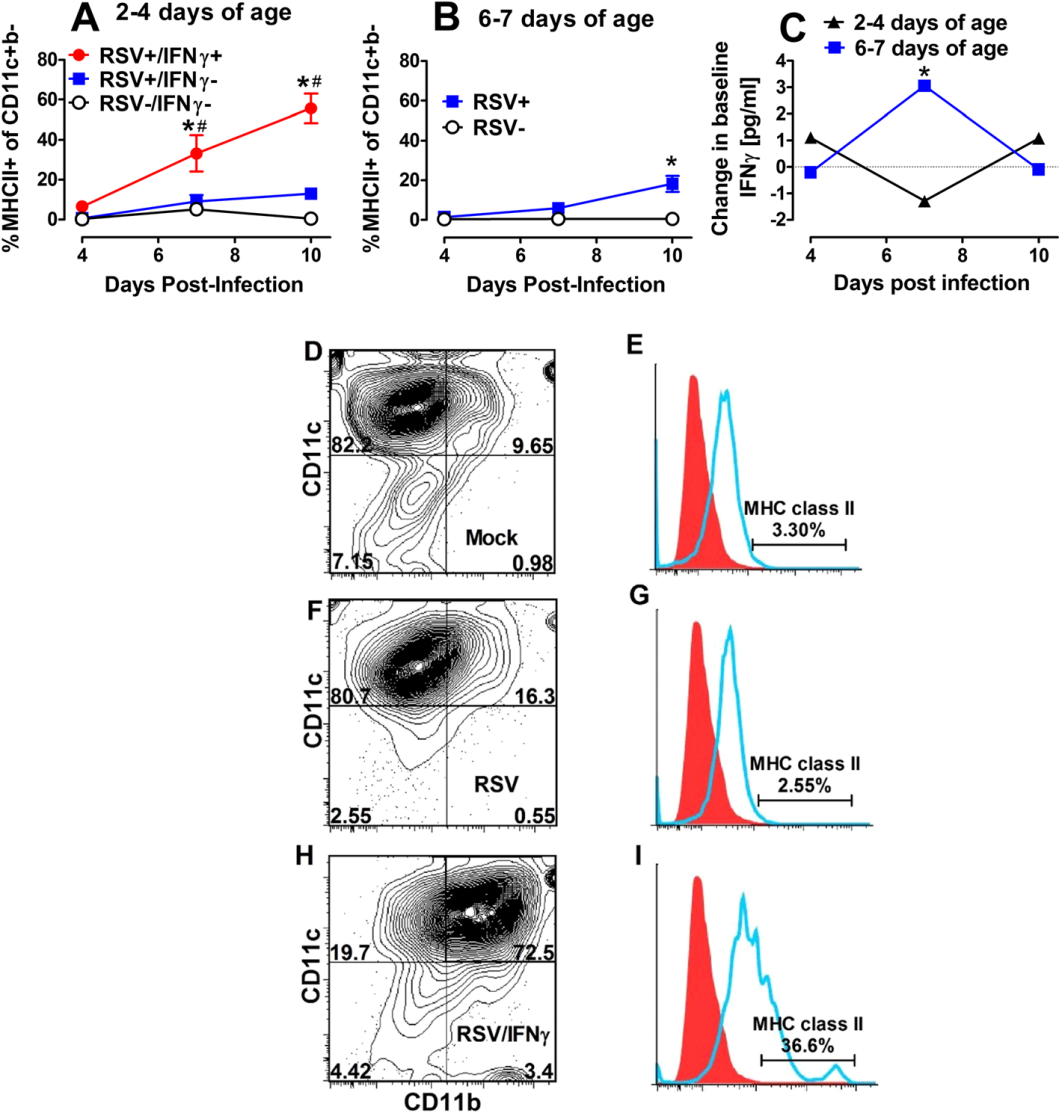
IFNγ exposure correlates with age-dependent AM activation. Neonatal Balb/c mice were infected with RSV L19 at 2 days (**a**) or 7 days (**b**) of age. Some mice were treated with 16 ng/g of i.n. IFNγ on 1, 3, and 5 dpi (**a**). BALF was harvested to quantify AM activation by flow cytometry (**a**-**b**) and IFNγ concentrations were measured and reported as the change from uninfected, age-matched controls (**c**). Data represent ≥ 5 mice per group and 2 separate experiments. * Indicates significant differences between RSV+ (+/− IFNγ) and RSV- groups and # between RSV+/IFNγ + and RSV+ groups based on a 2-way ANOVA with Bonferroni post-test; *p* < 0.05. Dot plots are representative samples from 7dpi, (the first time point in which MHC class II expression significantly increased in the RSV+/IFNγ + group) of at least five mice per group for RSV-/IFNγ- (**d**), RSV+/IFNγ- (**f**), and RSV+/IFNγ + (**h**). Each dot plot has a corresponding histogram representing MHC class II+ expression on CD11c + CD11b- cells with RSV-/IFNγ (**e**), RSV+/IFNγ- (**g**) and RSV+/IFNγ + (**i**) being presented

To determine the effect of age on RSV-mediated upregulation of MHC class II on CD11c + b- alveolar macrophages (AMs), cells were isolated from the BALF of BALB/c mice infected at different ages or treated with i.n. IFNγ. When compared to mock-infected, age-matched controls, AMs from mice infected at 2–4 days of age (Fig. 2a) did not become activated. However, when neonatal mice were infected at 6–7 days of age, MHC class II expression on CD11c + CD11b- cells significantly increased by 10dpi (Fig. 2b) suggesting that RSV-mediated AM activation depends on age at the time of infection. Moreover, the increase in MHC class II on CD11c + CD11b- cells was preceded by an increase in IFNγ concentrations in RSV- compared to mock-infected animals in BALF at 7dpi (Fig. 2c), which did not occur in neonatal mice infected at 2–4 days of age suggesting that RSV-mediated AM activation is age-dependent and may correlate with IFNγ.

The effects of i.n. IFNγ administration can be seen in the dot plots and histograms representing 7 dpi BALF samples. Large cells are characterized as CD11c + b- in mock-infected (Fig. 2d) and RSV-infected neonates (Fig. 2f). Neonatal AMs did not respond to RSV as evidenced by the lack of MHCII class II expression following infection (Fig. 2e, g). In contrast, RSV-infected neonates treated with i.n. IFNγ showed a marked shift in airway cells from CD11c + CD11b- to a larger population of cells that were CD11c + CD11b+, a population shown to have greater antigen presentation in the context of MHC class II expression and often characterized as dendritic cells (DCs) (Fig. 2h). In the RSV+/IFNγ + group, large cells that remained CD11c + b- also efficiently upregulated MHC class II+ expression (37 %) compared to PBS-treated controls (3 %) (Fig. 2i).

### Pharmacokinetic differences result in age-dependent AM activation

**Fig. 3:**
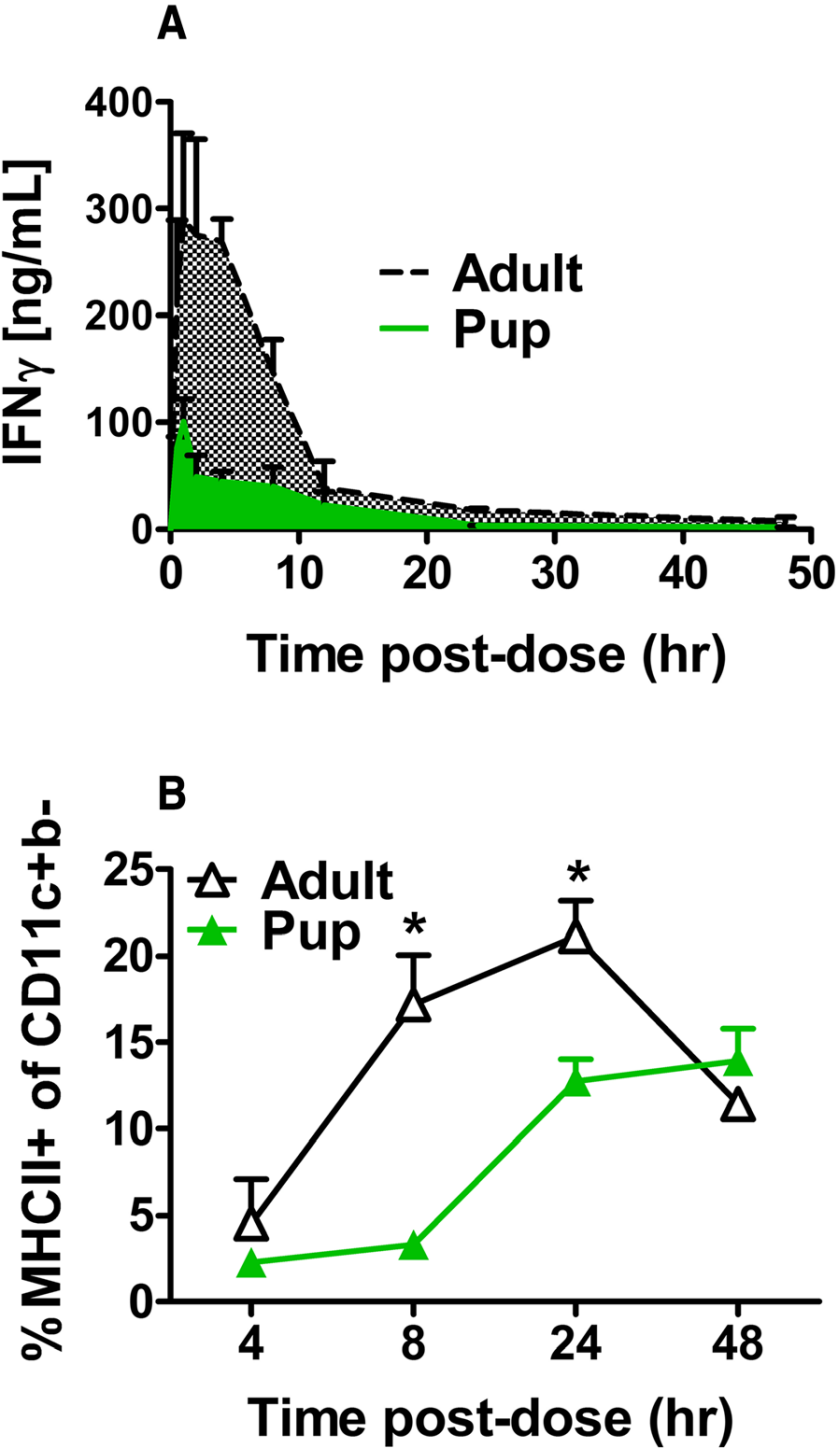
Age-dependent IFNγ pharmacokinetics result in differential AM activation. AUCs were determined for uninfected pup and adult BALB/c mice following a single i.n. dose of IFNγ (16 ng/g) through intense sampling from LD over 48 h (**a**). Biologically, this translated to significantly greater activation of AMs (**b**) in adults beginning at 8 h and continuing through 48 and 24 h, respectively. Data represent ≥ 3 mice per group and 2 separate experiments. * Indicates significant differences based on a 2-way ANOVA with a Bonferroni post-test, between groups at the indicated time points; *p* < 0.5

Based on the age-dependent associations between IFNγ exposure with AM activation and viral clearance, we next sought to determine the effect of age on the IFNγ area under the concentration-time (AUC) curve. Following a single 16 ng/g dose of i.n. IFNγ, AUCs averaged 3.6 times greater in adults *versus* neonatal mice (Fig. 3a). This was associated with significant increases in the expression of MHC class II on adult compared to neonatal CD11c + CD11b- cells through 48 h (Fig. 3b). To optimize neonatal IFNγ AUCs, a non-compartmental pharmacokinetic approach, as described in our methods, estimated a new 60 ng/g dose of i.n. IFNγ would be required to achieve adult-level AUCs (280 ng*hr/ml) in the neonatal mice. Thus, we predicted that 60 ng/g of i.n. IFNγ would generate faster and greater expression of MHC class II on CD11c + CD11b- cells, with corresponding enhancement of RSV clearance.

### IFNγ dose responsiveness correlates with improved AM activation and enhanced viral clearance

**Fig. 4:**
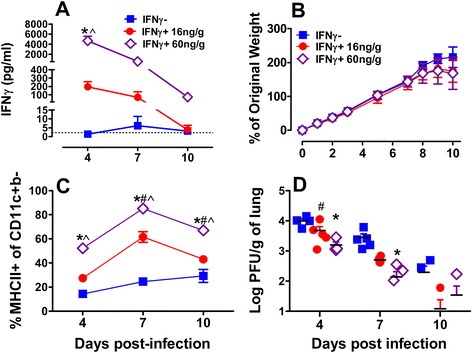
Neonates demonstrate dose dependent LM activation and viral clearance. Balb/c mice were infected with RSV L19 at 5–7 days of age and treated with i.n. IFNγ (16 or 60 ng/g) or PBS on 1, 3, and 5 dpi. Luminex was used to quantify IFNγ in BALF (**a**) and % of original weight was calculated from baseline litter weights (**b**). Lungs were collected for flow cytometry to analyze the % of CD11c + CD11b- cells expressing MHC class II+ (**c**) and viral titers using H & E plaque assay (**d**). # and * represent significant differences between pups treated with 16 and 60 ng/g compared to PBS treated pups, respectively. ^ indicates significant differences between pups treated with 16 vs. 60 ng/g of IFNγ using a 2-way ANOVA with Bonferroni post-test; *p* < 0.05

To test the effect of IFNγ dose on AM activation and viral clearance, RSV-infected neonatal mice receiving PBS only were compared to those treated with 16 or 60 ng/g of IFNγ. Significant differences in IFNγ BALF levels occurred as soon as 4dpi and dissipated over time reflecting the end of IFNγ dosing at 5 dpi (Fig. 4a). Despite its potential for toxicity, neither the 16 ng/g nor the 60 ng/g IFNγ groups demonstrated significant impairment in weight gain compared to PBS-treated controls over the course of the study (Fig. 4b), suggesting minimal pulmonary absorption. These findings are consistent with unpublished data generated in our lab showing negligible IFNγ levels in the serum of 4-day-old BALB/c mice following a single i.n. dose of IFNγ.

To determine the effect of IFNγ dose on total lung macrophage (LM) activation in RSV-infected neonatal lungs, the activation profile of neonatal LMs from LD was examined. At each time point tested, the 60 ng/g dose increased LM activation significantly more than pups treated with 16 ng/g or PBS alone (Fig. 4c). LMs from mice receiving 16 ng/g also significantly increased LM activation at 7 and 10 dpi compared to PBS-treated mice (Fig. 4c) but not at 4dpi, suggesting a stronger activation signal may be required for initial neonatal LM activation.

To determine the functional effect of optimizing i.n. IFNγ, RSV clearance was quantified by viral plaque assay. By 4 dpi, RSV clearance was greatest in the 60 ng/g group followed by the 16 ng/g of IFNγ compared to PBS; however, only the group receiving 60 ng/g demonstrated enhanced RSV clearance at 7 dpi (Fig. 4d). By 10 dpi, 80 % (4/5) of animals in both IFNγ groups had undetectable viral loads while only 50 % (2/4) of neonates in the PBS group had similarly undetectable virus.

### Low dose IFNγ is protective in the RSV-infected neonatal airway

**Fig. 5:**
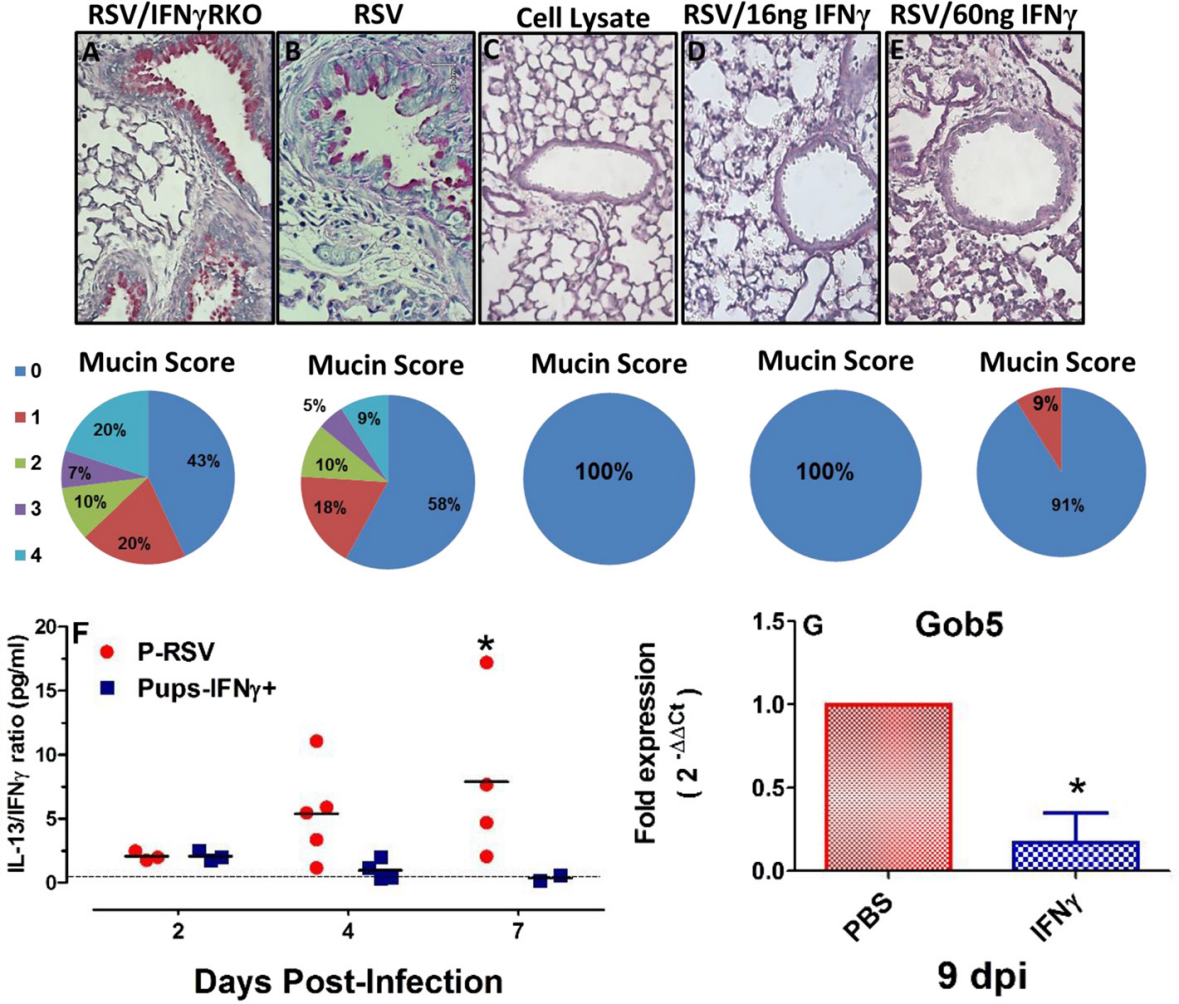
Local IFNγ reduces airway mucus production. WT and IFNγR KO pups were infected with RSV line 19 or cell lysate, then treated with i.n. IFNγ (16 ng/g or 60 ng/g) or PBS on 1, 3, and 5 dpi as described in the methods. The lungs were harvested at 8 dpi and sections compared by extent of PAS staining. Airways were scored 0 to 4 for PAS positivity according to previously published methods [23]. **a** Representative examples of airway mucin production from RSV/IFNγRKO (**a**), RSV (**b**), Cell lysate (**c**), RSV/16 ng/g IFNγ (**d**), and RSV/60 ng/g IFNγ (**e**). Pie charts are provided to numerically represent a percentage of the total airways ranked according to mucin scores. The ratio of IL-13 to IFNγ, detected in BALF, is given for RSV-infected pups treated with IFNγ compared to pups that received PBS only on days 2–7 post-infection (**f**). Lastly, Taqman RT-PCR, Gob5 expression was determined at 9 dpi in RSV-infected pups receiving i.n. IFNγ compared to PBS-treated pups (**g**). Data represent the means ± SD for 5 mice per group and 3 separate experiments

To determine the functional consequences of achieving higher, adult-like IFNγ AUCs in the neonatal airway during RSV infection, mucus production was determined in RSV-infected mice treated with 16 or 60 ng/g of IFNγ or PBS alone; IFNγRKO mice were included as an additional control group to assess mucus production in the absence of IFNγ signaling (Fig. 5a-e). Airway mucin scores were determined as previously described [23]. Mucus production was evident in representative RSV-infected neonatal lung sections (Fig. 5a, b). Conversely, mucus production was absent in uninfected neonatal mice (Fig. 5c), those treated with 16 ng/g of IFNγ (Fig. 5d) and mucus was markedly reduced in neonates that received 60 ng/g of IFNγ (Fig. 5e). Based on the role of IL-13 in mediating mucus production, the ratio of IL-13 to IFNγ was compared in neonates treated with i.n. IFNγ *versus* those receiving PBS only (Fig. 5f). As infection progressed, the ratio of IL-13 to IFNγ expanded such that by 7 dpi, IL-13 was significantly greater than IFNγ in RSV-infected neonates. However, the ratio remained balanced in IFNγ-treated neonates. The reduced expression of Gob5 in IFNγ-treated pups at 9 dpi compared to PBS-treated animals reiterates the importance of IFNγ in mitigating mucus overproduction in the RSV-infected neonatal lung (Fig. 5g).

**Fig. 6:**
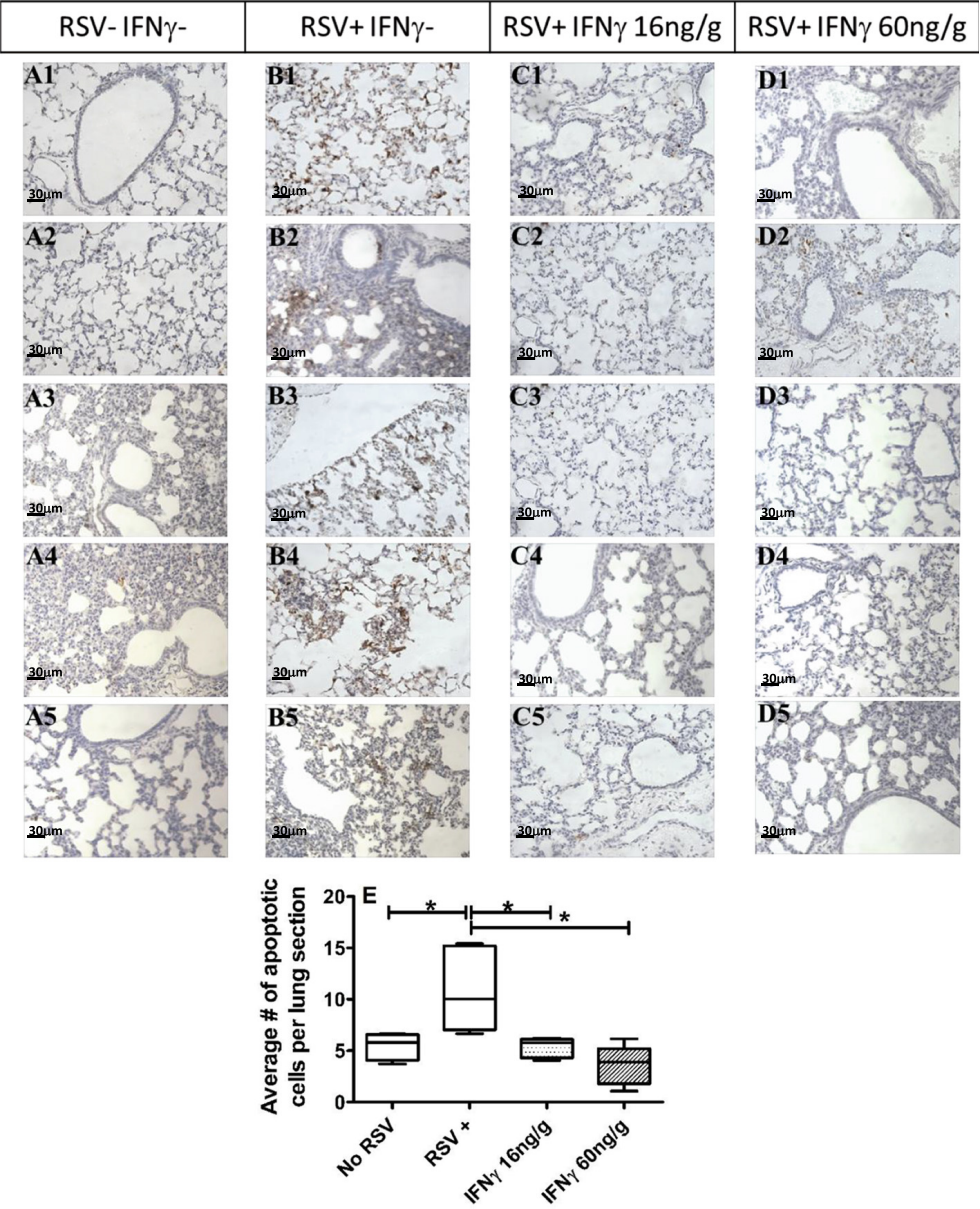
i.n. IFNγ reduces apoptosis in neonatal lungs at 8 dpi. Balb/c mice (5–7 days old) were infected with RSV L19 and treated with i.n. IFNγ (16 or 60 ng/g) or PBS on 1, 3, and 5 dpi. At 8 dpi right lungs were harvested, fixed with formalin and TUNEL stained. Two individuals blinded to treatment groups quantified TUNEL positive cells on each slide for each group. (A1-A5) Represents a single lung section from each animal in group A (RSV-/IFNγ-); Group B (RSV+/IFNγ-); Group C (RSV+ IFNγ 16 ng/g); and Group D (RSV+ IFNγ 60 ng/g). Images were captured at 40X magnification and the average number of apoptotic cells per lung section were quantified and graphed (E); there were 5 mice per group. * indicates a significant difference compared to RSV+ using ANOVA with a Tukey post-test; *p* < 0.5

To determine the effect of i.n. IFNγ on RSV-mediated apoptosis in the neonatal lung, TUNEL staining was performed at 8 dpi on lung sections from neonatal mice that were A) mock-infected; B) RSV-infected; C) RSV-infected and treated with 16 ng/g of i.n. IFNγ; or D) RSV-infected and treated with 60 ng/g of i.n. IFNγ (Fig. 6). Figure 6 shows representative lung section from each mouse per group. RSV-infected (Fig. 6b) lungs had significant increases in TUNEL positive staining compared to uninfected lungs (Fig. 6a). Interestingly, both 16 ng/g (Fig. 6c) and 60 ng/g (Fig. 6d) IFNγ-treated groups had reduced TUNEL staining compared to RSV-infected lungs at 8 dpi, which is graphically represented in Fig. 6e.

### Depletion of neonatal alveolar and lung macrophages using clodronate liposomes

**Fig. 7:**
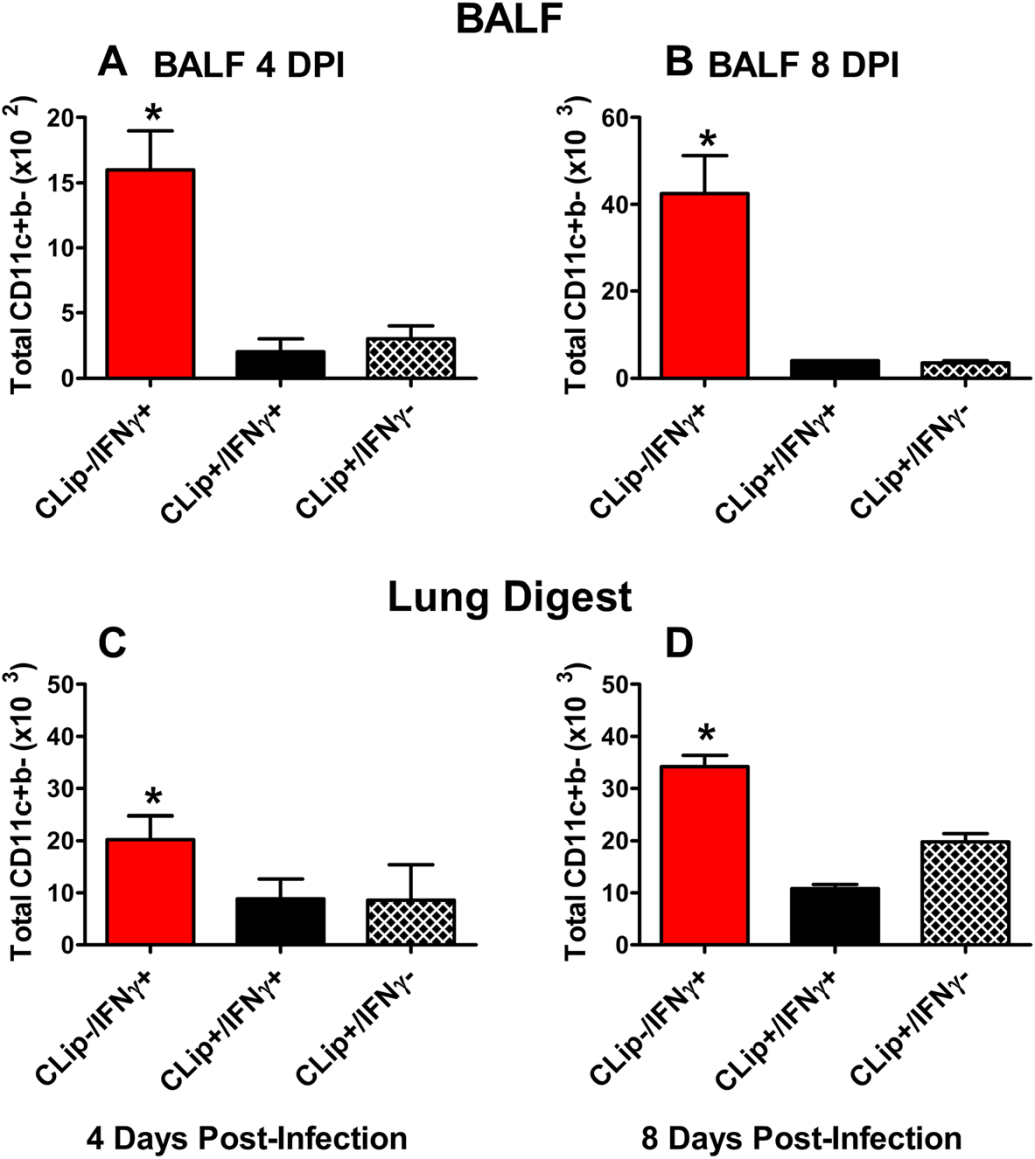
CLip effectively depletes AMs from RSV-infected neonatal lungs. Balb/c mice (2 days old) were treated with i.n. CLip and/or IFNγ as outlined in the Methods section. BALF (**a** and **b**) and digested right lung lobes (**c** and **d**) were harvested at 4 (**a** and **c**) and 8 (**b** and **d**) dpi. Total AMs (CD11c + CD11b-) were determined *via* flow cytometry. * Indicates a significant difference between groups using ANOVA with Bonferroni post-test. *p* < 0.5

Based on previously performed pilot studies in our lab, daily dosing of i.n. CLip was shown to sufficiently reduce the AM population in RSV-infected neonatal mice. CLip treatment began −1 dpi in 2 day-old mice; dosing continued daily with either CLip or PBS; pups then received i.n. IFNγ (16 ng/g) or PBS on days 1, 3, and 5 dpi. Regardless of i.n. IFNγ treatment, AM numbers in both the BALF and lung digest were significantly reduced with CLip at 4 and 8 dpi (Fig. 7). The AM population was reduced by 84 and 91 % at 4 and 8 dpi, respectively (Fig. 7a, b). Moreover, total LMs harvested from the LD were significantly reduced by 57 and 55 % at 4 and 8 dpi, respectively, following CLip treatment (Fig. 7c, d).

### IFNγ and AMs play a significant role in eradicating RSV from neonatal airways

**Fig. 8:**
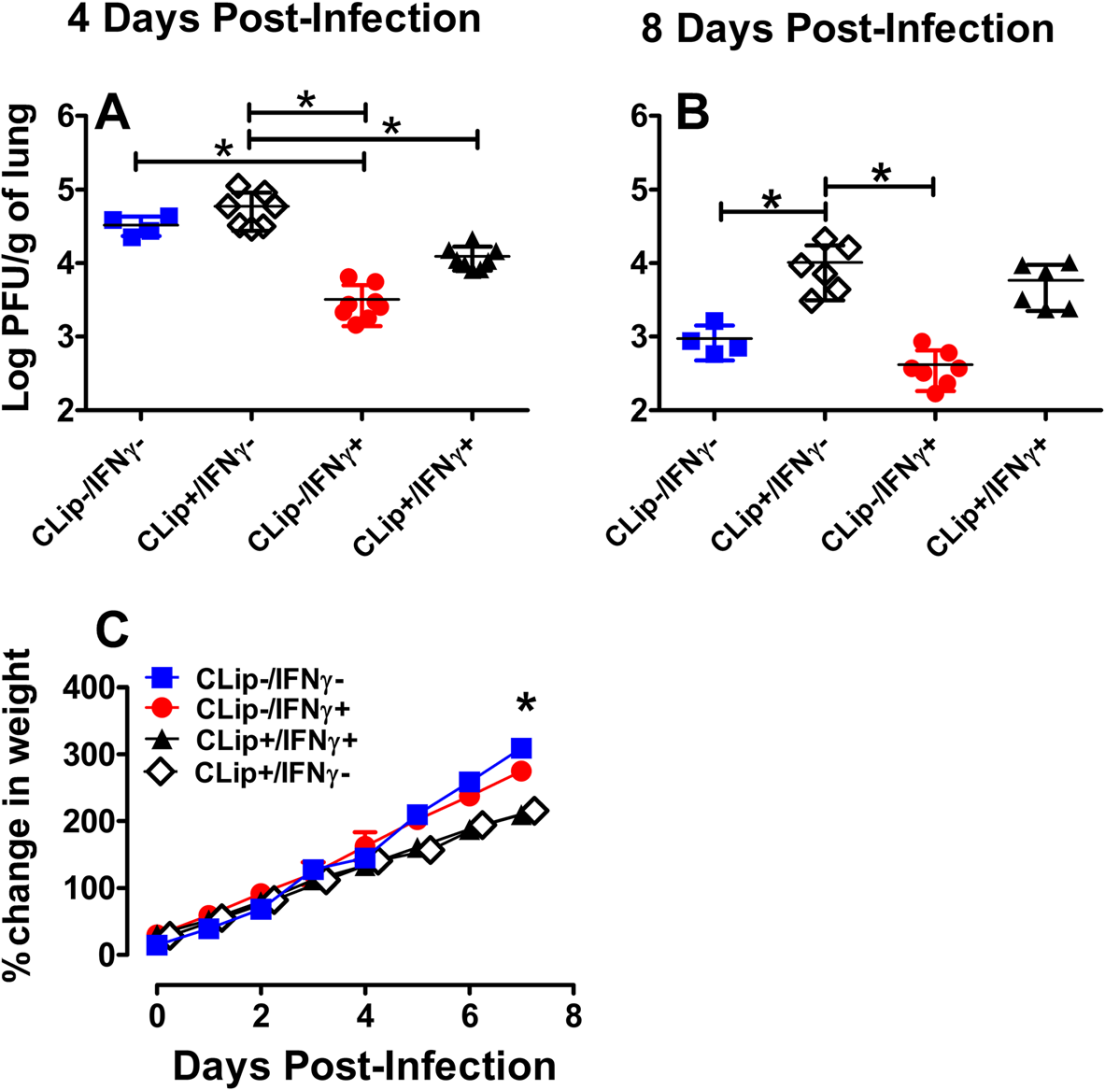
AM depletion reduces RSV clearance and impairs weight gain. Balb/c mice (2 days old) were treated with i.n. CLip and/or IFNγ as outlined in the Methods section. Left lungs were collected on 4 (**a**) and 8 (**b**) dpi for RSV quantification using plaque assay. Litters were weighed daily and the percent change in weight (**c**) was calculated from their baseline weight prior to i.n. treatments. **a** and **b** Means and individual replicates are depicted and statistical differences were defined as *p* < 0.05 using ANOVA with a Tukey post-test. **c** Mean ± SEM are depicted; *indicates a statistical difference between control (CLip-/IFNγ-) and CLip treatment groups (CLip+/IFNγ + and CLip+/IFNγ-) using repeated measures ANOVA (*p* < 0.05)

The functional effects of AM depletion and i.n. IFNγ treatment were tested following RSV infection *via* viral plaque assay. At 4 dpi, CLip (−)/IFNγ (+) animals had the lowest viral titers, which were significantly lower than CLip (−)/IFNγ (−) and CLip (+)/IFNγ (−) treated animals (Fig. 8a). The CLip (−)/IFNγ (+) group had lower viral titers than the CLip (+)/IFNγ (+) group but the difference was not significant. The CLip (+)/IFN (+) group had significantly lower viral titers than CLip (+)/ IFNγ (−) treated animals. These results suggest that IFNγ treatment played a larger role in reducing viral burdens than did depletion of AMs. To evaluate the contribution of each treatment independently, we analyzed viral titers by 2-way ANOVA. This analysis confirmed our hypothesis that IFNγ treatment was primarily responsible for the differences in viral titers. In fact, IFNγ treatment accounted for 45 % of the variability; however macrophage depletion also significantly contributed to differences in viral titers, accounting for 10 % of the variability. Bonferroni post-hoc analysis revealed that the significant effect of macrophage depletion (CLip treatment) was primarily a result of the differences in viral titers of CLip (+) vs. CLip (−) animals that were not treated with IFNγ.

At 8 dpi, CLip (+)/IFNγ (−) animals had the highest viral burden which was significantly higher than both CLip (−)/IFNγ (−) and CLip (−)/IFNγ (+) animals (Fig. 8b). However, viral titers of CLip (+)/IFNγ (−) animals were not significantly higher than CLip (+)/IFNγ (+) animals demonstrating that the viral clearing effects of IFNγ was lost by 8 dpi in the absence of AMs. In fact, treatment with IFNγ accounted for only 5 % of the variability observed. The presence or absence of AMs played a critical role in RSV clearance, accounting for 43 % of the variability. Regardless of IFNγ treatment, neonates whose AM populations were eliminated with CLip had RSV viral titers that were nearly a log fold higher than CLip (−) neonates at 8 dpi.

The potential for biological toxicity resulting from either CLip or IFNγ treatment was analyzed by calculating the neonates’ ability to gain weight over time. Interestingly, CLip treatment but not IFNγ significantly impaired the ability of neonates to gain weight (Fig. 8c). Visual inspection of the charted daily weights revealed a separation of the groups beginning at 4 dpi. Regardless of IFNγ treatment, the daily percent change in weight began to slow in CLip (+) neonates when compared to CLip (−) neonates beginning at 4 dpi and continued until the final time point. At 7 dpi, both CLip (+) groups demonstrated significantly impaired weight gain when compared to CLip (−)/IFNγ (−) neonates.

## Discussion

Since the formalin-inactivated RSV vaccine trials of the late 1960’s [22, 23], RSV-mediated pathology has been tightly linked to the host immune response [6, 13–15, 33]. Studies showing that ribavirin, a drug that blocks viral replication, does not effectively improve clinical outcomes such as hospital length of stay or time on mechanical ventilation [27, 38] has encouraged theories supporting RSV-mediated immunopathology. However, mounting evidence indicates that viral clearance kinetics plays an important role in disease severity [3, 4]. A recent study involving RSV-infected children < 2 years of age, demonstrated that a faster rate of RSV clearance was independently associated with shorter hospitalization [7]. Due to the fact that RSV replicates several times less efficiently in mouse models than in humans, debates continue over whether immature host immunity *versus* viral load contributes to severity of disease [4, 7].

We previously reported that i.n. IFNγ reduces the burden of RSV L19 in neonatal mice [23]. We further showed a preponderance of AMs in RSV-infected neonatal compared to adult airways throughout infection [23]. Based on our studies and those in human neonates demonstrating the dominance of AMs during RSV infection [19, 20], we tested the hypothesis that i.n. IFNγ enhances RSV clearance through activation of immature neonatal AMs. Using IFNγRKO mice, we showed that IFNγ significantly contributes to viral clearance in neonatal mice. To determine how immature AMs correlate with poor IFNγ exposure, we infected neonates at 2–4 or 7 days of age and analyzed AM activation in association with IFNγ production. In mice infected at 2–4 days of age, virtually no IFNγ production was detected which was consistent with an absence of AM activation [23]. Only when IFNγ was given exogenously did AM activation increase in this age cohort. However, when neonates were infected at 7 days of age, AMs became activated during RSV infection, though it was significantly delayed and reduced compared to RSV-infected adults. Moreover, it was preceded by a fleeting, but significant increase in IFNγ that was not observed in the younger cohort of mice indicating that in the neonatal lung, AM exposure to IFNγ was likely important for their activation.

Based on these age- and IFNγ-mediated increases in AM activation, we sought to better understand and immunokinetically manipulate the relationship between IFNγ exposure and AM activation in an effort to hasten viral clearance and in turn, minimize RSV-mediated pathology. Specifically, we examined the relationships between IFNγ exposure and its correlation with mucus production and apoptotic cellular debris among RSV infected neonates treated with i.n. IFNγ or PBS controls. We previously showed that expression of the suppressive cytokine TGF-β1, is upregulated in the lungs of neonatal compared to adult mice [30]. Therefore, in our immunokinetic analysis, we targeted a higher IFNγ exposure in neonatal animals than is typically observed, in anticipation of a lower AM activation threshold brought about, not only by RSV itself, but by elevated levels of TGF- β1. Additionally, it was assumed that neonates are equally efficient in their IFNγ signaling pathway as compared to adults, once IFNγ binds its receptor on AMs. This assumption was challenged by Marodi et al. who showed that IFNγ receptor-mediated signaling in neonatal macrophages harvested from cord blood compared to those from adult macrophages were reduced in regard to Candida killing, as well as in the release of superoxide anion [31]. By performing a pharmacokinetic analysis in which both neonates and adults received the same weight-based dose of IFNγ, we showed that the IFNγ AUC of adults was 3.6 times larger than that of neonates. Larger IFNγ exposure in the adults resulted in expedited and enhanced MHC class II expression on AMs in adults compared to neonates. The large age-based differences in IFNγ exposure was not a result of differential IFNγ metabolism but was most likely an artifact of the neonate’s larger lung to body size ratio.

Taking this into consideration, a non-compartmental model analysis was used to determine a new neonatal dose of IFNγ that would achieve more adult-level AUCs. We predicted the new neonatal IFNγ dose of 60 ng/g would not only expedite viral clearance, but would also modulate associated risk factors linked to skewed Th2 pathology, such as mucus production. The data presented here shows the capacity of IFNγ to induced AM activation and RSV clearance in a clear, dose-dependent manner without provoking weight loss. To determine the extent to which AMs contributed to RSV clearance, we then depleted neonatal AMs using clodronate liposomes, which further demonstrated that AMs significantly contribute to viral clearance in the neonatal lung.

Low levels of IFNγ in the BALF are consistently associated with greater disease severity in neonates [32] yet it is unclear what IFNγ’s protective role is. Consistent with this finding is data published by Cohn et al. demonstrating that mucus production is inhibited by IFNγ in an adult mouse model [33] and our previously published data showing greater mucus production in RSV-infected neonatal mice in which there is minimal IFNγ production compared to RSV-infected adult mice [23]. Consistent with previous findings, these data show here for the first time, reductions in goblet cell formation (as indicated by PAS staining) in RSV+ neonatal lungs treated with inhaled IFNγ (16 ng/g and 60 ng/g) when compared to untreated RSV+ pups. Mounting evidence continues to support the role of IFNγ as a regulator of mucus production [34–36]. Determining its precise mechanism in decreasing mucus production during neonatal RSV infection is an ongoing focus of research in our laboratory.

RSV non-structural proteins, NS1 and NS2, have been shown to impair early cellular apoptosis. It is speculated that this is advantageous to the virus; allowing RSV to spread from one cell to another throughout the airway [37]. Other data support the idea that RSV sensitizes cells to tumor necrosis factor-related apoptosis-inducing ligand (TRAIL)-mediated apoptosis by upregulating the expression of death-receptors 4 and 5 [38]. The sensitization is tempered however by early increases in Mcl-2, a member of the anti-apoptotic Bcl-2 family, which acts to delay the effects of TRAIL-mediated signaling and delays apoptosis in infected epithelial cells [39]. We reasoned, then, that IFNγ, a well-established and appreciated inducer of cellular apoptosis [13] may be associated with less severe RSV disease clinically [37], due to its facilitation of apoptosis thereby limiting the spread of the virus. To test the hypothesis that IFNγ interferes with RSV-mediated impairment of apoptosis, we examined the effect of i.n. IFNγ on apoptosis in RSV infected neonatal mice. Surprisingly, RT-PCR data showed that i.n. IFNγ elicited no change in caspase 3 or 8 expression by 4 or 7 dpi in RSV infected neonates treated with 60 ng/g of IFNγ compared to PBS-treated controls (*Unpublished data*), despite enhanced viral clearance. However, significant reductions in TUNEL-positive cells in groups receiving i.n. IFNγ suggest possible enhanced clearance of apoptotic cells rather than IFNγ-mediated changes in apoptosis. Studies are ongoing to determine the role of IFNγ in mitigating the clearance of apoptotic cellular debris in the neonatal airway.

We postulate that IFNγ may be augmenting mechanisms of viral resistance in airway epithelial cells that dramatically reduce the viral burden at the onset of infection. IFNγ treatment was independently associated with significantly lower viral titers at our earliest time point, 4 dpi, suggesting that IFNγ treatment reduced the initial viral burden. High levels of IFNγ exposure prior to RSV infection have been shown to dramatically inhibit RSV replication in adult BALB/c mice [40]. Additionally, exposure of a human airway epithelial cell line (A549) to IFNγ for 48 h prior to RSV infection reduced 2-day post-infection viral titers when compared to IL-4 treated or control cells. This reduction was associated with the upregulation of antiviral proteins, such as IFIT1 and Mx1 [41]. Ongoing studies are investigating the contributions of IFNγ-treated airway epithelial cells to reduced RSV infectivity.

Finally, to our knowledge this is the first study, using a neonatal animal model, to examine the role of AMs in the clearance of RSV through the i.n. administration of CLip. We demonstrated that AMs play a significant role in RSV clearance and that delayed viral clearance is associated with reduced weight gain in a neonatal murine model of RSV. Although AM-mediated regulation of weight gain requires further investigation, impaired weight gain in CLip *versus* PBS-treated pups highlights the importance of AMs during neonatal RSV infection. Mice do not develop clinical symptoms of RSV but a failure to appropriately gain weight suggests that the animals experienced detrimental effects from dramatically reduced viral clearance. As previously mentioned, clinical data from human neonates < 2 years of age found that faster RSV viral clearance was associated with shorter hospitalizations [5]. Future studies will investigate the histological effects of AM depletion in RSV-infected neonatal mice. We hypothesize that neonatal mice depleted of AMs have an abundance of necrotic debris and mucus obstructing their airways.

We have provided phenotypic and functional analysis of the role of IFNγ in neonatal RSV infection and furthered the understanding of AMs’ role in neonatal disease. These studies demonstrated the age-dependent development of AMs and the direct correlation of IFNγ in the neonatal AM response during RSV infection. Specifically, neonatal AMs display acute IFNγ dose responsiveness whereby higher doses of IFNγ are capable of overcoming the delay between IFNγ exposure and AM activation observed with lower IFNγ doses. Histological analysis of neonates infected with RSV and treated with IFNγ revealed the crucial role that it plays in reducing mucus production and decreasing the number of apoptotic cells in the airway. Lastly, AMs play a significant role in RSV clearance. Impaired RSV clearance as a result of AM depletion correlates with poor weight gain in neonatal mice. These findings warrant further investigation into the AM-dependent and independent effects of IFNγ in neonatal RSV disease.

Together these data indicate that early IFNγ exposure is critical to both reductions in viral burden and prevention of severe pathology in the neonatal airway. These data further demonstrate that i.n. IFNγ plays a critical role in reducing RSV burden early in infection and that neonatal AMs are crucial in coordinating efforts to eliminate RSV and return neonatal lungs to homeostasis.
